# Use of eye-tracking to evaluate human factors in accessing neonatal resuscitation equipment and medications for advanced resuscitation: A simulation study

**DOI:** 10.3389/fped.2023.1116893

**Published:** 2023-03-16

**Authors:** Linda Gai Rui Chen, Brenda Hiu Yan Law

**Affiliations:** Department of Pediatrics, University of Alberta, Edmonton, AB, Canada

**Keywords:** infant, newborn, neonatal resuscitation, human factors, eye-tracking

## Abstract

**Introduction:**

Emergency neonatal resuscitation equipment is often organized into “code carts”. Simulation studies previously examined human factors of neonatal code carts and equipment; however, visual attention analysis with eye-tracking might further inform equipment design.

**Objectives:**

To evaluate human factors of neonatal resuscitation equipment by: (1) comparing epinephrine preparation speed from adult pre-filled syringe vs. medication vial, (2) comparing equipment retrieval times from two carts and (3) utilizing eye-tracking to study visual attention and user experience.

**Methods:**

We conducted a 2-site randomized cross-over simulation study. Site 1 is a perinatal NICU with carts focused on airway management. Site 2 is a surgical NICU with carts improved with compartments and task-based kits. Participants were fitted with eye-tracking glasses then randomized to prepare two epinephrine doses using two methods, starting with an adult epinephrine prefilled syringe or a multiple access vial. Participants then obtained items for 7 tasks from their local cart. Post-simulation, participants completed surveys and semi-structured interviews while viewing eye-tracked video of their performance. Epinephrine preparation times were compared between the two methods. Equipment retrieval times and survey responses were compared between sites. Eye-tracking was analyzed for areas of interest (AOIs) and gaze shifts between AOIs. Interviews were subject to thematic analysis.

**Results:**

Forty HCPs participated (20/site). It was faster to draw the first epinephrine dose using the medication vial (29.9s vs. 47.6s, *p* < 0.001). Time to draw the second dose was similar (21.2s vs. 19s, *p* = 0.563). It was faster to obtain equipment from the Perinatal cart (164.4s v 228.9s, *p* < 0.027). Participants at both sites found their carts easy to use. Participants looked at many AOIs (54 for Perinatal vs. 76 for Surgical carts, *p* < 0.001) with 1 gaze shifts/second for both.

Themes for epinephrine preparation include: Facilitators and Threats to Performance, and Discrepancies due to Stimulation Conditions. Themes for code carts include: Facilitators and Threats to Performance, Orienting with Prescan, and Suggestions for Improvement. Suggested cart improvements include: adding prompts, task-based grouping, and positioning small equipment more visibly. Task-based kits were welcomed, but more orientation is needed.

**Conclusions:**

Eye-tracked simulations provided human factors assessment of emergency neonatal code carts and epinephrine preparation.

## 1. Introduction

Neonates may require resuscitation both at birth and in the Neonatal Intensive Care Unit (NICU) (1, 2). Specific neonatal resuscitation tasks are performed by trained healthcare professionals (HCP) using specialized equipment such as prefill epinephrine syringes, umbilical venous catheters, sterile trays, endotracheal tubes, and laryngoscopes. For ease of access, this equipment may be kept together in emergency equipment carts (“code carts”). However, in high stress situations, equipment in these carts may not be intuitive to find. Locating equipment is further complicated by the different weight and size-based choices specific to neonates, which may range from <500 g to >4 kg; size and weight adds mental load and complexity to equipment selection and is an equipment issue unique to pediatrics (3). Finally, some of this equipment, such as the prefilled emergency epinephrine kits, were designed for adult use and requires additional steps for neonatal use.

Previous studies in NICUs and pediatric units demonstrated that integrating human factor principles and considering both neonatal and pediatric resuscitation algorithms in the design of equipment carts can improve patient safety, decrease treatment delay, and increase HCP preference (3–5). One common theme in improving human factors include colour coding and cognitive aids to help choose size and weight appropriate treatments (3, 6). Local quality improvement initiatives have attempted to optimize code cart organization and improve healthcare provider (HCP) education surrounding the use of this equipment (7). Simulation have been used to analyze the performance of resuscitation equipment carts (4, 5); however, wearable eye-tracking glasses, tracking a person's eye movement and visual attention, might provide more information to inform future design, organization, and education of this equipment.

In this study, we aimed to analyze the physical ergonomics of neonatal resuscitation equipment, specifically how equipment design and organization may impact performance, user experience, and search time. Using a multi-modal simulation-based approach, we aim to evaluate two aspects of emergency neonatal resuscitation equipment including 1) emergency epinephrine preparation and 2) emergency equipment cart usability. Specifically, we used a combination of mobile eye-tracking, interviews, and surveys to provide rich data of the HCPs' user experience.

## 2. Methods

**Figure 1:**
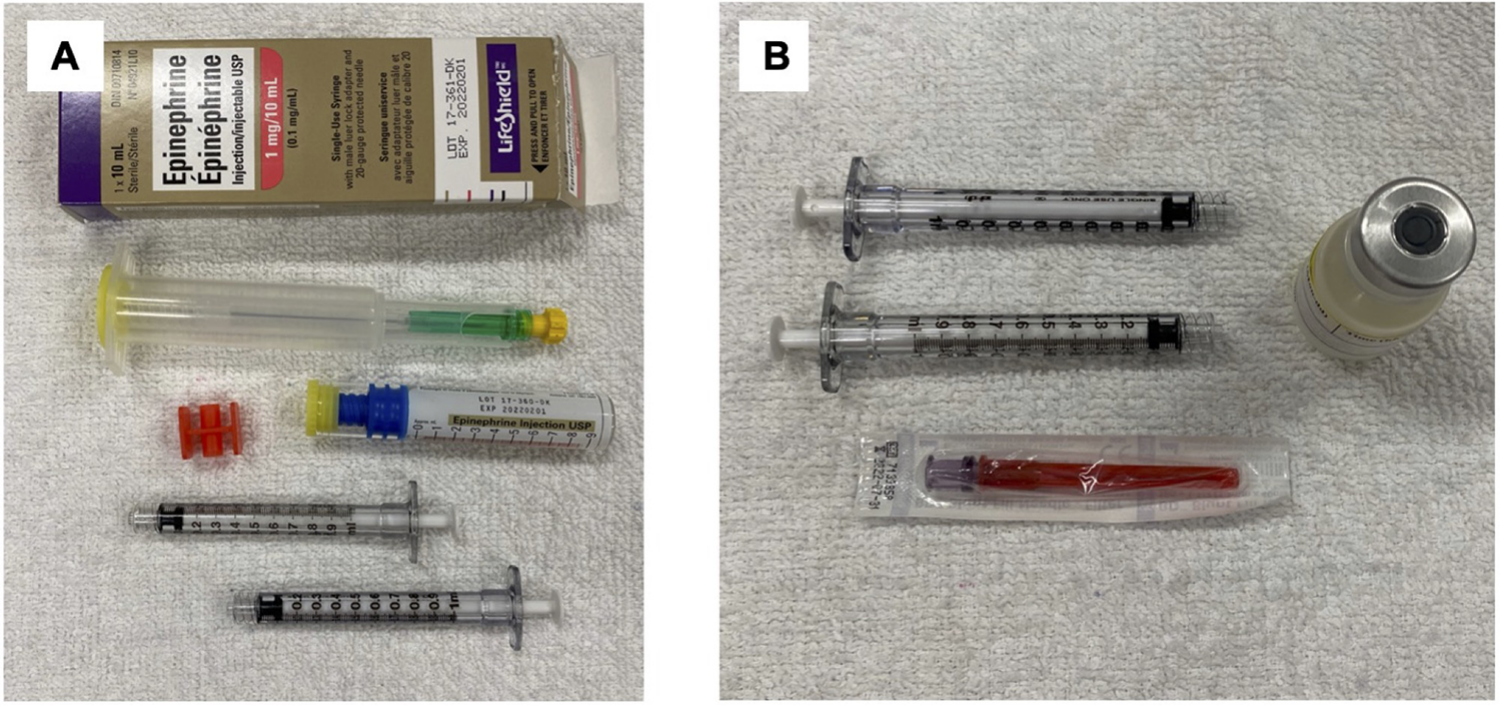
Epinephrine adult prefilled syringe for neonatal doses (**A**) vs. Simulated Multi-access Vial (**B**).

**Figure 2:**
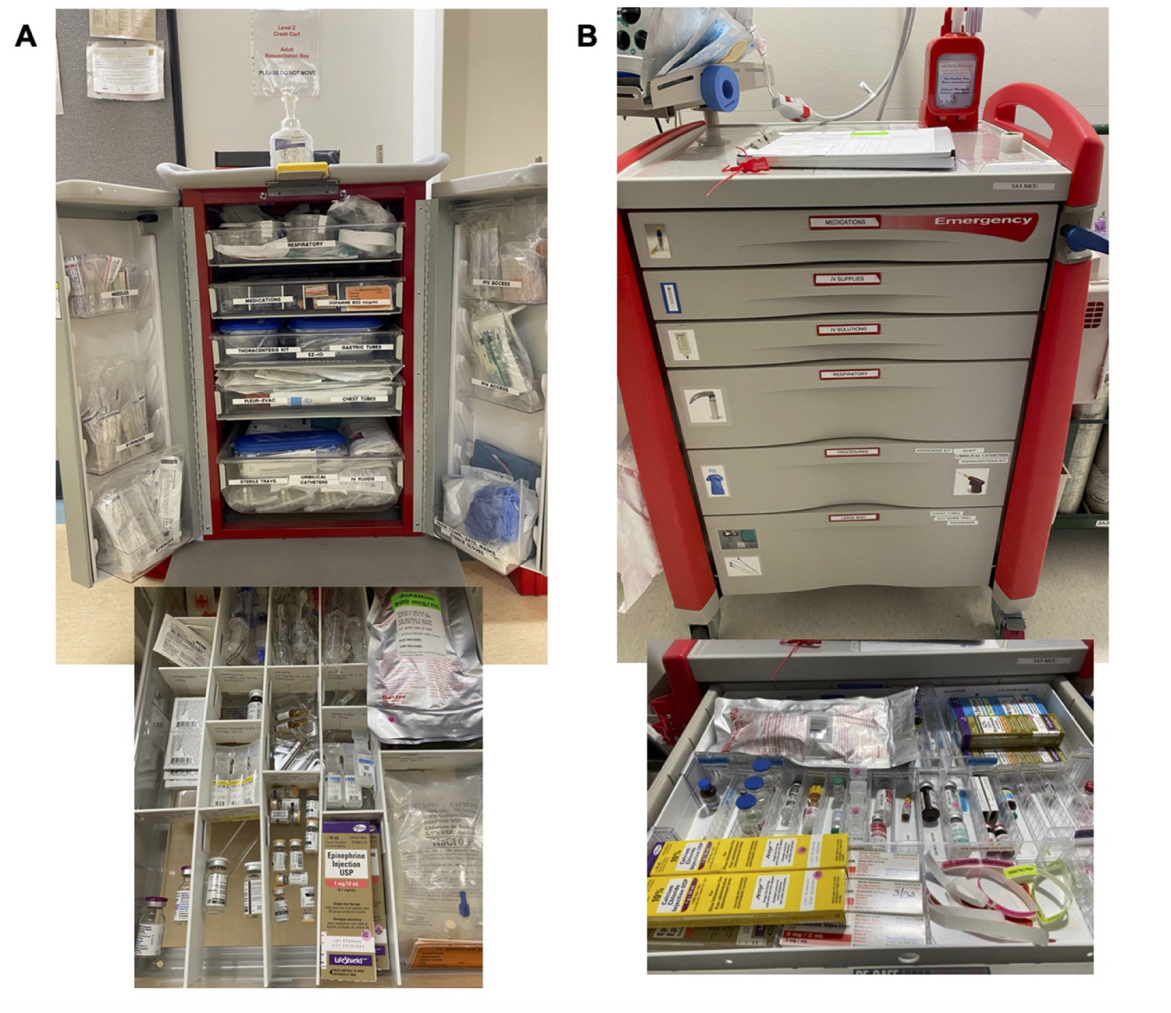
Neonatal emergency equipment carts and medication drawers for perinatal (site 1) (**A**) and surgical (site 2) (**B**) NICUs.

We conducted a randomized cross-over simulation study at two hospitals within one neonatal program. Site 1, Royal Alexandra Hospital, is a high-volume level 3 NICU located in a regional perinatal center admitting preterm infants 22 weeks and above and older infants needing significant neonatal intensives care including total body cooling, ventilation, vasoactive supports etc (“Perinatal Site”). The code cart at Site 1 has an emphasis on airway management but contains equipment for medications, intravenous access, and specialized procedures. Site 2, University of Alberta Hospital, is a level 4 NICU located at a children's hospital and is a regional referral center specializing in infants with surgical and complex needs, such as infants with critical congenital heart disease, congenital diaphragmatic hernia, abdominal wall defects, airway anomalies, etc. (“Surgical Site”). The code cart at Site 2 was designed to for response to peri-operative and non-perioperative emergencies, improved with better compartment organization and task-based procedure kits. There were two main objectives: (1) to compare two methods of emergency epinephrine preparation using a cross-over design for all participants (Figure 1) and (2) to compare two different code carts across the two sites (Figure 2). While each NICU has a different code cart design, both carts contain similar equipment with only minor differences (e.g., in specific medications stocked other than epinephrine) (Figure 2). Both code carts have undergone recent evaluation for optimization, with the process for redesigning the Surgical code cart previously described (7). The Health Ethics Research Board, University of Alberta (Pro00110698) approved the study.

Participants were recruited from available on-duty personnel at each NICU using convenience sampling. Physicians, nurse practitioners, advance practice nurses, registered nurses, and registered respiratory therapists were eligible to participate, provided they worked clinically in that NICU. Individuals were excluded if they declined to participate or if they could not be fitted to the eye-tracking glasses. Recruitment occurred from June to August 2021. After written informed consent has been obtained, participants were fitted with eye-tracking glasses (Tobii Glasses 2, Tobii Technology, Inc.), which recorded an approximation of the participant's visual attention (8, 9). First, emergency epinephrine preparation was tested. Participants were randomized to draw up two resuscitation epinephrine doses (0.01 mg/kg) using two different methods, either starting with either an adult epinephrine prefilled kit or a multiple access vial simulating same epinephrine concentration (Figure 1). Randomization was performed *via* seal opaque envelops prepared by an assistant not involved in data collection, using a computer-generated random number sequence. Two different patient weights were used for each method, and participants had access to weight-based emergency drug dosing sheets as a reference (available on both code carts as standard practice for the units, which pre-calculates doses and volumes for emergency drugs including epinephrine.) Participants were told that we wanted to see how fast they could prepare these doses, that they were timed, and therefore to do the task as fast as they could.

Second, finding and retrieving equipment from the code cart was tested. Participants were given 1 min to look over the cart at their site prior to the start of the simulation. As our aim was not to test participants under stress, this orientation gave all participants the same minimum pre-simulation exposure to their respective equipment carts. Pre-task orientation also allowed us to ask questions about how participants oriented themselves to the cart after the simulation. Finally, it allowed us to focus on the equipment organization rather than the physical mechanics of the cart (i.e., how the doors / drawers opened) which we could not change. They were then asked to obtain items for seven procedures using the code cart at the unit where they were recruited. These tasks include: (1) intubation (laryngoscope, stylet, 3.0 endotracheal tube), (2) intravenous insertion (24 gauge catheter, saline flush, connector), (3) umbilical venous catheter insertion (UVC kit, 5Fr single lumen umbilical catheter), (4) epinephrine administration (prefilled epinephrine kit, 1 ml syringe, rapid fill connector) (5) adenosine administration (adenosine 3 mg/mL, gummy connector or 3 way stop cock) (6) thoracocentesis procedure (thoracocentesis kit and 10.2 Fr pigtail chest tube) and (7) nasogastric tube insertion (8 Fr tube).

After the tasks were completed, we sought feedback from each participant for both tasks (epinephrine and code cart). First, participants' experiences were assessed through a post-simulation electronic survey. Then, an eye-tracking augmented semi-structured interview was conducted, following an interview guide (Appendix A). Each participant was asked to provide a running commentary while viewing their own eye-tracked video-recording of their performance. This approach was previously used in analyzing eye-tracked videos of neonatal resuscitation (10). The videos were also paused at various points, where semi-structured questions were asked, particularly if the participant seemed to have problems performing a task. Both researchers participated in the first 5 interviews to ensure that the format worked well, then one researcher (LC) completed the remaining interviews independently. Interviews were audio recorded for later data analysis.

**Figure 3:**
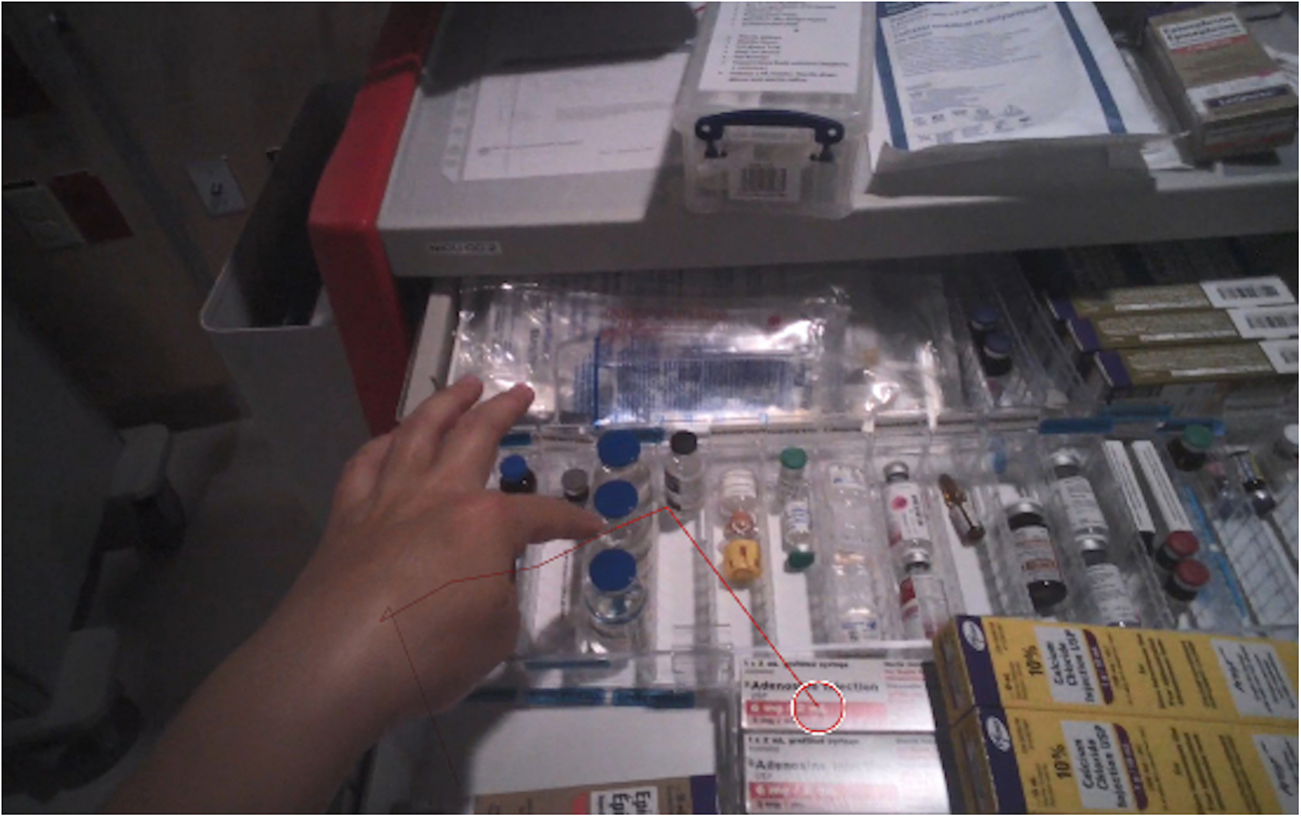
Still image of eye-tracking during equipment search (circle indicates current visual attention, lines delineating gaze shifts).

Eye-tracking videos were used to analyze objective performance measures (Figure 3). Time from request to two doses of epinephrine ready to administer were compared between the two epinephrine techniques. Time to obtain equipment for all seven tasks, as well as time to obtain equipment for each individual tasks were compared between the participants at the two sites. Using a combination of manual and computerized analysis (Tobii Lab Pro, Tobii Technology, Inc.), visual attention was analyzed for the equipment retrieval task, from the when the first item was requested to when the last item was retrieved from the cart. Visual attention was quantified by measuring (a) number of different areas of interest (AOIs) and (b) frequency of gaze shifts between areas of interest.

Interviews were subject to thematic analysis. First, interviews were transcribed verbatim using a combination of automatic electronic transcription (www.rev.com) with subsequent manual checking (LC). Both research team members then familiarized themselves with the transcripts through repeated re-reading. Researchers then independently applied a descriptive coding scheme before meeting to discuss and formulate themes and subthemes related to each task, informed by concepts in human factors and cognitive psychology.

### 2.1. Sample size and statistical analysis

A pre-determined pilot sample of 40 participants (20 at each site) was chosen. This was sufficient to allow for recruitment from different disciplines at each site as well as a sufficient sample size for eye-tracking data based on previous studies (8, 9). Previous eye-tracking studies performed at this center had 75%–88% of recordings of sufficient quality for analysis (8, 9); assuming a ∼75% rate, a sample of 40 recordings would yield ∼30 recordings that can be analyzed for quantitative VA data. This provides a sample for VA comparable to other eye-tracking simulation studies (*n* = 20–30) (8, 11).

Data is presented as mean [standard deviation (SD)] for normally distributed continuous variables and median [interquartile range (IQR)] for skewed distributions. Correlations were determined using Pearson's correlation coefficient. For the speed of epinephrine dose preparation repeated measures ANOVA was used to compare the two methods, using randomization (which method used first) as a between subjects factor to account for possible crossover effect. Means were compared using Student's t-tests for parametric data and medians were compared using Mann-Whitney-U test for non-parametric data. Categorical and nominal data were compared using Fisher's Exact Test. Statistical analyses were performed with SPSS 27 (IBM, Armonk, New York, USA).

## 3. Results

### 3.1. Part 1. Quantitative results—task speed, surveys, and visual attention

Forty HCPs participated (20 from each site), including nurses (*n* = 25, 65%), respiratory therapists (*n* = 5, 12.5%), neonatal nurse practitioners (*n* = 5, 12.5%) and physicians (*n* = 5, 12.5%). Distribution of disciplines were similar between the two sites. All participants completed the simulation and interviews per protocol. (Table 1). Seven other HCPs were approached but declined to participate. One individual could not be fitted with the eye-tracking glasses (could not obtain data when wearing the eye-tracking glasses over corrective glasses) and was excluded prior to starting any simulations.

**Table 1:** Summary of participants and quantitative results.

Participants		Site 1 (Perinatal)	Site 2 (Surgical)	Total
Number of Participants	20	20	40
Discipline	Registered Nurses	12 (60%)	13 (65%)	25 (62.5%)
	Respiratory Therapists	2 (10%)	3 (15%)	5 (12.5%)
	Nurse Practitioners	4 (20%)	1 (5%)	5 (12.5%)
	Physicians	2 (10%)	3 (15%)	5 (12.5%)
**Epinephrine Preparation**		**Multiple Access Vial *median (IQR)***	**Prefilled Syringe *median (IQR)***	***P* value**
Time to Prepare (seconds)	Both doses	52.8 (43.7–62.5)	64.3 (54.4–90)	<0.001
	First dose	29.9 (24.7–36.6)	47.6 (35.5–66)	<0.001
	Second dose	21.2 (17.2–25.7)	19 (15.8–25.3)	0.563
**Code Cart Task**		**Site 1 (Perinatal) *median (IQR)***	**Site 2 (Surgical) *median (IQR)***	***P* value**
Time to obtain code cart items	All items	164.4 (149.8–218.4)	228.9 (193–297.1)	0.027
	Intubation	20.8 (18.7–32.8)	50.1 (41.4–60.5)	<0.001
	PIV start	19.6 (15.9–35.8)	19.9 (15.1–29.3)	0.752
	UVC insert	19.9 (14.5–23.2)	15.6 (13.7–22.3)	0.114
	Epinephrine	18.6 (15.4–25)	15.5 (12.1–17.9)	0.343
	Adenosine push	10.7 (8–12.1)	47.5 (29.2–86)	<0.001
	Thoracocentesis	24.7 (16.6–27.1)	21.9 (19.2–37.1)	0.752
	NG insert	7.7 (4.6–12)	9 (7–18.7)	0.752
Visual Attention During Code Cart	Number of Distinct AOIs visited	54 (49–57)	76 (66–86)	<0.001
	Gaze shifts Frequency (per second)	1 (0.7–1.1)	1 (0.9–1.1)	0.443
**Post Simulation Surveys**		**Site 1 *n (%)***	**Site 2 *n (%)***	***P* value**
Survey Responses (Agree/ Strongly Agree)	I prefer the epinephrine prefilled syringe	9 (45%)	8 (40%)	0.452
	Cart was easy to use	17 (85%)	18 (90%)	1.0
	Equipment was easy to find	17 (85%)	18 (90%)	1.0

It was faster to draw the first epinephrine dose using a standard medication vial (29.9s vs. 47.6s, *p* < 0.001), but time to draw the second dose was similar (22.5 vs. 21.6, *p* = 0.563). As a result, it was faster to obtain both doses using a standard multiple access vial compared with the prefilled syringes (52.8 vs. 62.3, *p* < 0.001). Randomization (which method was used first) had no effect for either first dose or total time, denoting a lack of cross-over effect. Most participants (87.5%, 35/40) took more time to prepare the first dose using the prefilled syringe.

It was faster to obtain equipment for all 7 tasks from the Perinatal cart (164.4 vs. 228.9s, *p* = 0.027). This difference is the result of longer time to obtain intubation equipment (20.8 vs. 50.1, *p* < 0.001) and longer time to obtain adenosine equipment (10.7 vs. 47.5, *p* < 0.001). PIV access equipment and adenosine administration equipment took the longest to find at the Perinatal and Surgical sites respectively. Time to obtain equipment were similar for all other tasks.

All participants completed the post-simulation survey. Despite the epinephrine prefill syringe being standard at both NICUs, less than half of participants preferred the prefilled syringe over the multiple access vials (45% vs. 40%). Participants at both sites found their respective carts easy to use (85% vs. 90%, *p* = 0.452) and equipment easy to find (85% vs. 90%, *p* = 0.452).

Twenty-six eye-tracking recordings (65%) were of sufficient quality and analyzed for quantitative visual attention metrics. Eye-tracking videos were analyzed if they had fixation data for at least 75% of the recording and if a pre-analysis manual screening demonstrated reliable data (i.e., no significant missing data during the tasks and points of fixation can be easily identified with minimal noise). Areas of interest for the carts were defined as individual distinct compartments or areas of the cart that a participant would look at, for example a well-delineated and separated section of a drawer or a bundle of equipment such as endotracheal tubes. Eye-tracking analysis revealed complex visual search patterns for the equipment; participants looked at many different areas of interest (54 for Perinatal vs. 76 for Surgical, *p* < 0.001) and had 1 gaze shifts per second for each task at both sites.

### 3.2. Part 2. Qualitative interview results

#### 3.2.1. Task 1: preparation of emergency epinephrine

##### 3.2.1.1. Theme 1: facilitators to performance

Participants describe what helped expedite their medication preparation process. Subthemes included experience, cues and prompts, low response effort, and low cognitive effort (Table 2).

**Table 2:** Summary of themes from interviews.

Task	Theme	Subtheme
Epinephrine Preparation	Facilitators to Performance	Experience
		Low Response Effort
		Low Cognitive Effort
		Cues and Prompts
	Threats to Performance	Inexperience
		High Response Effort
		High Cognitive Effort
		Deviations
		Violations
	Discrepancies due to Stimulation Conditions	-
Code Cart Access	Threats to Performance	Inattentional Blindness
		Indistinct Pieces
		Clutter
		Look-alikes
		Competing Schema
		Recognizable but Unhelpful Schema
		Inexperience
	Facilitators to Performance	Aids and Prompts
		Helpful Understanding of the Schema
		Experience
	Orienting with Prescan	-
	Suggestions for Code Cart Improvement	-

###### 3.2.1.1.1 Experience

HCP reported using past experience derived from (i) specific “experience using that prefilled syringe” or (ii) from generalizable areas such as adult codes (e.g., “back to my days in emerg where we would just shove the whole thing in there”) or (iii) using similar equipment (e.g, “it's something that you do with other medications, not just epinephrine”). Participants also reported confidence and knowledge in using the prefilled syringe and/ or the medication vial with statements such as “It's a system that I'm familiar with. So I stick with this.”.

###### 3.2.1.1.2 Low response effort

Participants described how equipment design resulted in a lower amount of required effort to complete the task, also known as “response effort”. For example, (i) easier physical set up (e.g., “I find the mechanism of using… the vial is faster because there's less connecting parts”) and ii) less resulting obstacles, such as bubbles, were brought up when describe their use of medication vials.

###### 3.2.1.1.3 Low cognitive effort

HCPs described lower cognitive load due to (i) feelings of safety for the prefilled syringe and (ii) less steps to recall for the vial. Safety was derived from built-in equipment safeguards for practitioners and knowledge of sterility/ safety for the patient (e.g, “I'm not risky poking myself or anybody else.”) when using the prefilled syringes. Easy step recall was described as “you don't have to remember all the other stuff. It's just put the needle on and pull up the dose, it's simpler.”.

###### 3.2.1.1.4 Cues and prompts

Participants described referencing visual cues like “hav[ing] to remove every yellow piece” from the prefilled syringes and using written instructions.

##### 3.2.1.2. Theme 2: threats to performance

HCPs described possible threats and barriers to performance, resulting in nonstandard protocol or delays in treatment. Subthemes included inexperience, high response effort, high cognitive load, deviations, and errors.

###### 3.2.1.2.1 Inexperience

Some HCPs reported lack of practice resulting in slower equipment set up and overall performance of drawing up medication. While standard, many participants mentioned that they “don't use the preloaded syringes very often”. Some participants mentioned that more practice would have made the experience easier.

###### 3.2.1.2.2 High response effort

HCPs frequently described a high response effort required for drawing up the epinephrine dose. Specifically, participants had difficulty with (i) equipment set up (e.g., it's stressful because there's a lot of parts and pieces) and (ii) physical obstacles due to equipment issues (e.g., “I'm drawing and it's not actually like filling my syringe. It was just air, which was annoying me”.

###### 3.2.1.2.3 High cognitive effort

High cognitive effort such as (i) multiple step recall, (ii) safety/ sterility concerns were common. Many participants described “trying to remember…what to connect, where” during the setup process, resulting in delay. There is also the added mental load of paying attention to personal safety; many participants had concerns of needle stick injury (e.g., “there's a risk of poking yourself… in a real code, sometimes people have the shakes and you could accidentally poke yourself.”). HCPs also had concerns of equipment sterility, and resulting delay in care (e.g, “because really you should be swabbing for like 20 s and then letting it dry. So then that's 40 s…you're just waiting. It's pretty significant delay”).

###### 3.2.1.2.4 Deviations

Potential deviations from proper technique stemmed from lack of experience with equipment, or simply never having learned it. This was particularly prominent with the adult prefilled epinephrine syringe. Some participants “just never learned on the rapid fill” and thus used an unsafe workaround by “default(ing)…[to stabbing the vial with] needles rather than [using] the quick connect.”.

###### 3.2.1.2.5 Violations

Particularly with the adult prefilled epinephrine syringe, some participants found more intuitive and lower response effort techniques which were deliberate violations of equipment's intended design. Some HCPs described that “in real life I probably would have just stuck the needle into the glass vial” instead of using the rapid fill connector “if we”re just being fast”.

##### 3.2.1.3. Theme 3: discrepancies due to stimulation conditions

Most participants felt that simulation conditions contributed to nerves (e.g., “this is where I get really nervous. Somebody watching me”). Some participants were also thrown off by differences in simulation conditions compared to real life, describing events such as “I felt really weird because I was like, these aren't in packages and I knew that it wasn't like sterile”. As well, as equipment were reused, occasionally malfunctions would occur in simulation, causing distraction and delay [e.g., “I feel like I would have had less trouble with (equipment) had it been a new thing.”].

#### 3.2.2. Task 2: code cart access

##### 3.2.2.1. Theme 1: threats to performance

Participants described perceived threats to their performance, including: Inattentional blindness, Indistinct pieces, Clutter, Look-a-likes, Competing schema, Recognizable but unhelpful schema, lack of standardization, Response effort, and Inexperience (Table 2).

###### 3.2.2.1.1 Inattentional blindness

HCPs frequently described inattentional blindness, resulting in pieces “hiding in plain sight”. Many noted the actual location was “very obvious…if you have an idea of what you're looking for.”.

###### 3.2.2.1.2 Indistinct pieces

Participants reported confusion over indistinct pieces of equipment, due to inadequate labeling, with participants noting that “the labels don't tell you exactly what everything is. It's a generic label” or there simply being “no label”. Manufacturer labels on packaging were also reported to be small and difficult to find, especially on tubing. Another source of difficulty was poor visibility due to stacking and clutter, awkward orientation (e.g., “looking…down…at 180 degrees” for labels), and placement of small pieces in the back of drawers.

###### 3.2.2.1.3 Clutter

Clutter and extra sizes of equipment were significant hindrances to performance. HCPs reported that because “there's just so much”, equipment often falls out or result in “jammed drawers”. Bags and kits were also described as “bloated” and “and required the entire container to be emptied before searching through for desired pieces. This clutter is worsened by having “so many of each [tube]”, with further delay from “weeding through the sizes” for the requested one.

###### 3.2.2.1.4 Look-alikes

Common items causing confusion included similar looking medication boxes (e.g bicarbonate and epinephrine), stacked kits, and bundles of equipment such as chest tubes. This was misleading, as some participants “assumed the 2 boxes [epinephrine and bicarbonate] were the same thing” and looked elsewhere for desired pieces. The similarity in packaging dimensions and design, as well as the close proximity of straight and pigtail chest tubes also mislead some participants. Participants mentioned it was common to grab the wrong medication box or chest tube by mistake. During the simulations, many participants were noted to glance at or physically pick up all similar looking items to double check for correctness.

###### 3.2.2.1.5 Competing schema

Equipment was often stored in unexpected locations. Many participants reported expecting a different schema of equipment organization than implemented in the carts [e.g “It doesn't make sense that it's over (there)”]. The most common complaint being “It's hard when things are separated for the same procedure…when you're looking at two separate drawers”. As well, some Site 1 participants noted a delay in retrieving medications, as the medication drawer seemed to start alphabetically but did not follow the same pattern towards the middle. Many participants understood the reasoning behind the organization “in hindsight”, but also reported that “it definitely wasn't [their] first thought”.

###### 3.2.2.1.6 Recognizable but unhelpful schema

Participants reported that the lack of standardization within the cart and throughout the ward contributed to delays in their search. Specifically, variations in (i) organization across equipment carts (e.g., “I'm not used to it being on the left side…cause I'm used to grabbing it from our drawer and…it's on the right”), (ii) design/ labeling of packaging (e.g “everyone labels their stuff differently. Every manufacturer”), and iii) terminology (e.g., “ I didn't know the extension tube was called T connector”) were threats to performance.

###### 3.2.2.1.7. Response effort

Inappropriate response effort was also the cause of performance delays. (i) low effort required for non-desired objects often confused participants [e.g., the 2.0 ETTs “shouldn't be the first thing in the drawer to see when…standard ETT tube is a three or three and a half, (which) should be more accessible.], typically side-tracking HCPs into the wrong compartment. Conversely, (ii) high response effort was often required for desired objects [e.g “if a baby (needs adenosine)…I now have to open a box too and then run through to find what I need”]. This mismatch of desired behaviour and response effort required resulted in avoidable search difficulties.

###### 3.2.2.1.8 Inexperience

Another threat to performance stemmed from lack of experience (e.g., “we don't have to use that often”) and education, with the “neonatal world [being] new” for some participants. However even for experienced HCPs, unfamiliarity with (i) available equipment (e.g., “I didn't know we had a connector”), (ii) available kits (e.g., “I didn't even know we had an adenosine kit”), and (iii) packaging (e.g., “the pigtail looks different in the package then it does in the baby”) was prominent. Many also reported that the respiratory drawer was “a little bit trickier for no other reason other than it's…the drawer I would go in the least”, as respiratory needs were usually handled by RTs with their own equipment kits.

##### 3.2.2.2. Theme 2: facilitators to performance

###### 3.2.2.2.1. Aids and prompts

Some HCPs reported aids and prompts facilitating their search, including (i) physical tools like a “ready-made” kit (e.g., “ it's a one-stop shop when you pick up that package”), (ii) visual cues like pictures on the drawers, prominent labeling, and easy to spot pieces themselves (e.g., “I could see it sticking out…the little orange feeding tube) (iii) extra stimulus prompts like “bright colours” (e.g., “the yellow [highlighting] made [finding the tube guage] somewhat less of a process”) and “highlighting”, (iv) written/ textual prompts (“I liked the labels cause then I would know I'm in the right space”), (v) positional prompts, such as having the desired object “right at the front”, and (vi) the Von restorff effect, where a distinct stimulus stands out (e.g., “it's so out there that you're like, I'm going to remember this one”).

###### 3.2.2.2.2 Helpful understanding of the schema

An understanding of the schema behind the organization and design of the cart was important in expediting the search process (e.g., “there”s nowhere else I can think of looking for it” or “I actually expected it there”). Some participants also reported having a “mental map” of the cart. This general mental layout helped them reason out where unfamiliar equipment may be kept (e.g., “So I guess that's a framework in my mind already that I generally know where things are based on how other things are structured.”). Specifically, participants appreciated equipment being organized by task: “I like the fact that everything is here, you don't have to collect…the flushes and the extension tubing and everything”.

###### 3.2.2.2.3 Experience

HCP described experience being a major facilitator in code cart usage. Visual familiarity with equipment and packaging helped HCPs know “exactly what [they were] looking for”. This familiarity often came from (i) nonspecific experiences like “stock[ing] the code cart and check[ing]”, and “teaching NRP”. HCPs also attributed success to (ii) specific experiences such as watching their colleagues (e.g “I knew where it was because I had seen people struggle with where it is”), simulation practice, and team roles (e.g “Since I became LC, I use a code cart more than I've ever done as a nurse”).

##### 3.2.2.3. Theme 3: orienting with prescan

Participants were given one minute to look over the cart before the simulation began. In this time, participants reported scanning for (i) categories and themes (e.g “ just to kind of make sure I had a general sense of like what groupings were there”), (ii) cues and prompts (e.g., “how the drawers are labeled to know what to expect inside”, (iii) unfamiliar equipment (e.g., “ I was just quickly making sure I knew some of the contents of the drawers I don't know as well”) (iv) general drawer content (e.g., “reminding myself, what's in every, in each drawer? “), and (v) to confirm existing knowledge (e.g., “I was just seeing if it was what I was used to. So if the things I'm normally used to seeing were in each drawer”).

##### 3.2.2.4. Theme 4: suggestions for code cart improvement

Participants suggested improvements to the code carts based both on their simulation performance as well as from past real-life experience. Suggestions for both code carts were similar. These included: (i) adding visual prompts and cues (e.g., color-coding, stickers and tabs, highlighting, and more prominent labeling), (ii) increasing visibility, (e.g., with smaller things in the front) (iii) grouping items by task, (iv) changing the organization schema (i.e organizing by size, by task, by frequency of use, or by alphabetical order), (v) increasing standardization (both within and between sites), (vi) reducing the amount of equipment (i.e., removing uncommonly used items, relocating items to outside the cart) and (vii) increasing education.

In particular, grouping equipment by task (i.e., having a “kit” for a specific procedure such as thoracocentesis or adenosine administration) was welcomed, but many participants needed more education or prompts on the contents of the kits, and some noted that the kits were difficult to find, despite external labelling and other visual cues. Some participants were very aware of the existence of different kits for different tasks, but unfamiliar with the contents of each kit and thus had difficulty retrieving specific pieces of equipment from within the kits.

## 4. Discussion

We successfully used a multi-modal approach with low complexity simulations to analyze the physical ergonomics of two neonatal resuscitation tasks: (1) preparing emergency epinephrine and (2) obtaining equipment from an emergency equipment “code cart”. The use of simulation, eye-tracking, surveys, and eye-tracking guided semi-structured interviews provided rich information on the usability of this equipment and highlighted facilitators and barriers of both tasks.

Emergency epinephrine for cardiac arrest (1:10,000, 0.1 mg/ml concentration) is not available in vials in the North American market; access is almost universally *via* adult-dosing prefilled syringes, which are colour coded to make epinephrine distinct from other commonly stocked adult resuscitation medications such as calcium and sodium bicarbonate. Epinephrine is also available in 1 mg/ml ampoules, which require dilution. Prefilled syringes for emergency medications are intended to (1) increase speed and ease of administration (12), (2) decrease risks associated with glass ampoules, (3) decrease risk of contamination, and (4) reduce medication error (e.g., using the wrong concentration of epinephrine or mix-up with another medication (13). However, neonatal resuscitation is distinct from adult and pediatric resuscitation in that medications other than epinephrine are rarely used and epinephrine boluses cannot be directly administered from the adult prefilled syringe due to the small doses and volumes (14). These limitations reduce the advantage of prefilled epinephrine syringes for the neonatal population. An additional step of drawing a neonatal dose into a separate syringe from the prefilled set requires additional connectors or stopcocks, which adds complexity and delay, as demonstrated in our study. Some participants found workarounds due to the complexity, which may in fact lead to unsafe use. Design of neonatal specific pre-filled epinephrine syringes of the 0.1 mg/ml concentration could decrease the complexity of this task, while maintaining the patient safety advantages.

Uniquely, eye-tracking provided both quantitative and qualitative data for our analysis, particularly for the code cart evaluations. First, quantitatively, the cart that took longer for participants to acquire equipment for all 7 tasks was also the code cart with more distinct areas of visual interest (i.e., number of compartments). Frequency of gaze shifts were similar between the two sites; therefore, this may indicate that increasing number of compartments increased the complexity for the visual search and may increase the time to equipment access for those who are unfamiliar with the code cart design. This represents a trade-off between organization distinctiveness and complexity and should be considered in future designs.

From a qualitative perspective, interviews conducted while viewing eye-tracked videos provided rich data of the user experience. By viewing the eye-tracked video of their performance, participants gave highly detailed accounts of their performance and provided specific feedback on equipment organization such as preferred organization schemes and factors that hindered their search. Other themes derived from these interviews readily conformed to known psychological and human factors principles such as response effort (15), mental workload (16), and inattentional blindness (17). These interviews also revealed how cues designed to help visual search may not be noticed or used by users, despite best intentions; when asked about labels and pictures on the front of the code carts, some participants did not place any visual attention on the labels, nor did they find the visual labels helpful. However, other participants suggested adding more labelling and visual cues to improve the code cart usability.

Finally, equipment design and organization cannot exist in isolation; user education and real-life experience a play a large role in facilitating success. For example, game-based simulation practice (a unit-wide “Simulation Olympics Competition” occurred at Site 1 months prior to the study where healthcare providers competed to be “fastest” at grabbing equipment) contributed to user knowledge and familiarity of their code cart and was referenced by some participants as contributing to their success. Familiarity obtained through checking and restocking code cart equipment also contributed to success. In contrast, advantage of task-based equipment kits for tasks such as UVC insertion, thoracocentesis, and adenosine administration were dampened by a lack of user knowledge about their existence and their specific contents. Thus, mechanism of education and familiarization should be incorporated to help optimize code cart access in unit such as the NICU, where carts may not be frequently accessed in real-life emergencies.

There were several limitations. First, participants completed tasks without the context of a realistic resuscitation scenario; in a simulated resuscitation, users may encounter more stress, workload, or distractions that could affect their performance and behavior. However, in our units, equipment access is usually designated to a single team member not tasked with other responsibilities. Second, problems associated with multiple users attempting to access the code cart could not be evaluated. Third, not all eye-tracking videos could be analyzed for visual attention due to missing data points; however, all eye-tracking videos were of sufficient quality for each participant to view during their interviews. Despite these limitations, eye-tracking provided rich data that could not have been obtained using video recording and simulations alone. Indeed, we demonstrated how visual attention analysis can be used both quantitatively and qualitatively to study human factors in the clinical environment, improving our understanding of tasks such as epinephrine preparation or retrieving equipment from code carts.

## 5. Conclusions

Eye-tracked simulations provided an in-depth assessment of human factors associated with emergency neonatal resuscitation equipment carts and equipment, revealing strengths and potential directions for improvement. While standard, adult prefilled epinephrine syringes were complex to use for the preparation of neonatal doses and could contribute to delays. Multi-modal data revealed facilitators and barriers to performance, and participants used their simulation experience to suggest specific code cart improvements which could be implemented to increase usability.

## Data Availability

The datasets presented in this article are not readily available because Video-recordings with participant identifiers will not be available. Requests to access the datasets should be directed to Brenda Law, blaw2@ualberta.ca.

## Supplementary material

Semi-structured Interview Guide
