# Supplementary material for: Use of eye-tracking to evaluate human factors in accessing neonatal resuscitation equipment and medications for advanced resuscitation: A simulation study
Source: Front Pediatr. 2023 Mar 16;11:1116893. doi: 10.3389/fped.2023.1116893 (PMC10060515; doi:10.3389/fped.2023.1116893)
Supplement: Semi-structured Interview Guide [file Table1.docx]

**Semi-Structured Interview Guide**

**A. Epinephrine**

As we watch the videos, tell me what you are thinking as you are doing the tasks. I may stop you to ask questions.

(If the person is struggling with the task) Tell me about the problems you are having at this moment. What might have made this easier?

(After both tasks are finished) Tell me about your experience with both methods.

**B. Code Cart**

As we watch the videos, tell me what you are thinking as you are doing the tasks. I may stop you to ask questions.

(If the person is struggling with the task) Tell me about the problems you are having at this moment. What might have made this easier?

(If the person is struggling to find a piece of equipment) Tell me where you expected this piece of equipment to be. How might we make it easier to find?

(If the person found the equipment quickly) Tell me why you were so quick to find this piece of equipment

(After the simulation) Tell me about your overall experience with the code cart.
